# Immune Memory-like Responses of Akoya Pearl Oyster to Pinctada Birnavirus

**DOI:** 10.3390/pathogens15040380

**Published:** 2026-04-01

**Authors:** Tomomasa Matsuyama, Kousuke Umeda, Takashi Atsumi

**Affiliations:** 1 Japan Fisheries Research and Education Agency, Pathology Division, Aquaculture Research Department, Fisheries Technology Institute, Minami-Ise 516-0193, Mie, Japan; 2 Mie Prefecture Fisheries Research Institute, Shima 517-0404, Mie, Japan

**Keywords:** Pinctada birnavirus (PiBV), pearl oyster, *Pinctada fucata*, immune memory-like responses, trained immunity, reinfection, transcriptome analysis, mantle

## Abstract

Among accumulating knowledge of invertebrate immune-like responses, antiviral mechanisms in mollusks remain poorly understood. Pinctada birnavirus (PiBV) infects the mantle epithelial cells of the pearl oyster (*Pinctada fucata*), causing mass mortality of juveniles during high-temperature periods. Here, we examined immune memory-like responses of pearl oysters to PiBV reinfection. At a high temperature (24–25 °C), experimental infection caused ~80% mortality, whereas mortality remained <30% and did not differ significantly from uninfected controls at a lower temperature (18–20 °C). Juveniles that survived infection at the low temperature and were subsequently reinfected at the higher temperature showed 10% mortality, which was significantly lower than the ~50% observed in naïve oysters infected under the same conditions. At 28 days post-infection at the lower temperature, oysters exhibited gene expression profiles distinct from those of naïve oysters. Ex vivo infection demonstrated significantly reduced PiBV replication in mantle explants from previously infected oysters compared with those from naïve individuals. These findings indicate that *P. fucata* acquires resistance to PiBV reinfection, and at least part of this resistance is mediated within the mantle, independently of other tissues. Culture supernatants of mantle explants from previously infected oysters were positive for viral genomic RNA even without viral inoculation, suggesting that persistent infection may contribute to the maintenance of immune-like responses.

## 1. Introduction

Invertebrates lack antigen-specific clonal expansion of lymphocytes that underlies immune memory in vertebrates, and it was previously believed that their defense against pathogens relies solely on non-specific innate immunity [1]. However, recent studies have reported phenomena resembling immune memory in various invertebrate species [2]. These immune-like responses in invertebrates, which differ fundamentally from vertebrate adaptive immunity, are often referred to as “immune priming,” “trained immunity,” or “innate immune memory” [3]. Although not clearly defined, these phenomena are generally explained as the ability of the innate immune system to respond to previously encountered infections or non-self-stimuli and maintain enhanced responsiveness to re-stimulation over the long term.

Immune memory-like responses in invertebrates have been extensively studied in crustaceans and some insects such as bees, which are economically important species, with the aim of protecting them from infectious diseases [4,5,6]. In mollusks, immune-like responses to bacteria or bacterial components have been reported in gastropods such as abalone, and bivalves including clams, mussels, and oysters [7]. In contrast, research on antiviral responses in mollusks is largely limited to studies on ostreid herpesvirus 1 (OsHV-1) and haliotid herpesvirus 1 (HaHV-1), which belong to the family Malacoherpesviridae in the order Herpesvirales [8,9,10,11,12,13]. This is partly because only a few viral diseases in shellfish are available as experimental models [14]. Expanding the research scope to include viral pathogens other than herpesviruses will contribute to a comprehensive understanding of antiviral immunity in bivalves.

In Japan, mass mortality of pearl oysters (*Pinctada fucata* (Gould)) due to viral disease has occurred since 2019, leading to a decline in pearl production. According to statistics from the Ministry of Agriculture, Forestry and Fisheries of Japan, annual pearl production, which had remained stable at 20–21 tons from 2010 to 2018, dropped to 13 tons by 2021. Juvenile mortality occurring in periods of high temperatures is characterized by atrophy of the soft body; hence, this condition is referred to as “summer atrophy” [15]. The causative agent of this disease is an unclassified birnavirus that likely forms a new genus closely related to the genus Entomobirnavirus and has provisionally been named Pinctada birnavirus (PiBV) [16]. PiBV infects epithelial cells on the outer surface of the mantle, causing cell destruction and detachment of the mantle from the nacreous shell layer, resulting in dorsal retraction of the soft body. Although PiBV causes high mortality in juveniles, infections in adults result in much lower mortality. Surviving individuals develop symptoms of “shell disease,” characterized by melanin deposition in the nacreous layer [17]. The optimal temperature for replication of PiBV is 25 °C, and multiplication of the virus is drastically reduced at 15 or 32.5 °C [18]. Consequently, outbreaks occur when water temperatures exceed 20 °C, with high mortality rates during the summer season [19,20].

Based on PiBV infection experiments and field observations, the presence of immune memory-like phenomenon against viruses is also suggested in the pearl oyster. In in vivo experimental infections at 25 °C, PiBV loads peaked at 2 days post-infection (dpi) with detachment of infected epithelial cells [16]. By 4 dpi, viral loads fell to about one-tenth of the peak with no remaining histopathological abnormalities. By 21 dpi, PiBV became undetectable in three of four individuals. These observations suggest that oysters that had overcome this transient replication acquired the ability to suppress PiBV re-replication, and this assumption is supported by field survey results. Specifically, Hashimoto et al. [19] found that juveniles that had experienced infection during spring when water temperatures were around 20 °C, exhibited lower mortality during re-infection in the summer (at >25 °C) compared to naïve cohorts that encountered the virus for the first time. The pearl oyster is phylogenetically closely related to the Pacific oyster (*Magallana gigas*), as both belong to the order Ostreida [21], and is therefore a suitable species for comparative studies on immune-like responses. In addition, because birnaviruses have markedly different virological characteristics from those of OsHV-1, the most extensively studied pathogen in bivalve immunology, studying infections caused by PiBV provides a valuable model for a wider understanding of antiviral defense mechanisms in bivalves.

**Figure 1:**
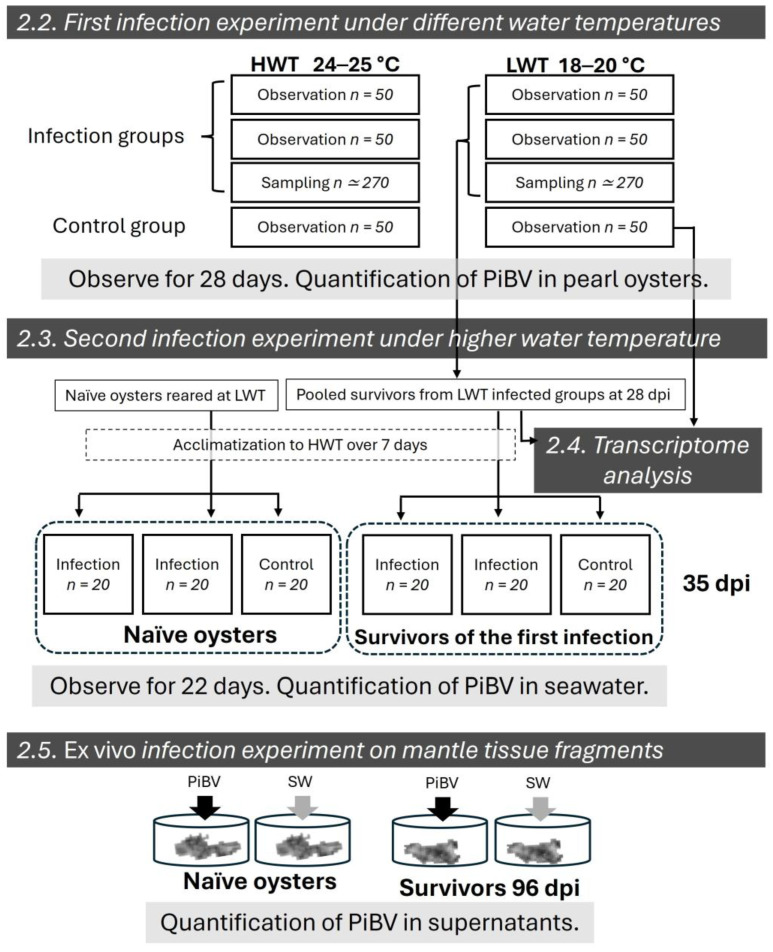
Experimental design.

In this study, a series of experiments were conducted as shown in Figure 1. To experimentally reproduce the immune memory-like responses against PiBV observed in field studies [19,20], an infection study was conducted at different water temperatures, and survivors from the low-water-temperature infection treatment, where cumulative mortality was low, were subjected to a second infection at higher water temperature, where higher mortality was expected. Additionally, transcriptome analysis was performed for comparative transcriptome profiling between the previously infected and the naïve groups to characterize the condition of the group that had acquired resistance. Furthermore, an ex vivo infection for mantle fragments was conducted to verify whether immune memory-like responses occur at the site of PiBV infection.

Schematic shows treatment groups in experiments described in Section 2.2, Section 2.3, Section 2.4 and Section 2.5 in Materials and Methods. Briefly, the first infection test was conducted at higher water temperature (HWT, 24–25 °C) and lower water temperature (LWT, 18–20 °C) over a 28-day period with monitoring for mortality, mantle atrophy, and PiBV levels. Transcriptome analysis was performed on the LWT control group and survivor group collected at 28 days post-infection (dpi). For the second infection trial, surviving oysters from the first infection under LWT and naïve oysters were acclimated to HWT over 7 days. The trial started at 35 days after first infection, and mortality and PiBV levels in seawater were monitored for 22 days. Finally, mantle tissues from survivors of the first infection experiment at 96 dpi and naïve juveniles from the same batch as the survivors were cultured ex vivo. These tissues were inoculated with either PiBV or seawater, incubating for 7 days, and quantifying the genome copy number of PiBV in the supernatant.

## 2. Materials and Methods

### 2.1. Pearl Oysters

Hatchery-produced juvenile pearl oysters were reared at the Mie Prefectural Fisheries Research Institute in land-based tanks supplied with filtered seawater until transport to the laboratory at the Japan Fisheries Research and Education Agency for use in experiments. The oysters were maintained in 65 L tanks filled with 56 L of seawater flowing at approximately 100 mL/min. The seawater had been filtered through a 1-μm pore filter and subsequently irradiated with UV light. Salinity and pH of the seawater were 3.1–3.3% and 8.1–8.2, respectively. The tanks were maintained either at LWT (18–20 °C) or HWT (24–25 °C) under aeration. The oysters were kept under natural photoperiod conditions and fed cultured diatoms (*Chaetoceros neogracile*, approximately 5 × 10^5^ cells/mL) at a rate of 10 mL per 1 L of tank water, five times per week, both during the acclimation period and throughout the infection experiments. Details regarding oyster size and the specific tank volumes used during the infection trials are provided in the respective experimental sections. Unless otherwise specified, the experimental conditions during the infection trials remained identical to those described above.

### 2.2. Preparation of PiBV

PiBV suspensions used as the infection sources were prepared as described in a previous study [18]. Briefly, PiBV was propagated in ex vivo-cultured mantle tissue, purified from the culture supernatant using CsCl density gradient centrifugation, and subsequently dialyzed into autoclaved seawater. The PiBV suspension was supplemented with penicillin G and streptomycin sulfate at final concentrations of 1000 units/mL and 1000 μg/mL, respectively. The mixture was then filtered through a 0.2 μm filter (Thermo Fisher Scientific, Waltham, MA, USA) and stored at 4 °C until use. Different batches of the PiBV suspension were used in each experiment.

### 2.3. First Infection Experiment at LWT and HWT

Juvenile oysters with shell length ranging from 6.3 to 10.2 mm (mean ± standard deviation [SD]: 7.7 ± 1.2 mm) and wet weight, including shells, ranging from 21 to 70 mg (40.5 ± 17.2 mg) were used. These values were calculated based on measurements from 50 randomly selected oysters. For LWT and HWT treatments, approximately 370 acclimated juveniles were transferred into a plastic container containing 100 mL of autoclaved seawater at the corresponding temperatures. The number of oysters was estimated based on the total weight and the mean individual wet weight.

A PiBV suspension was added to each container (PiBV: 4.4 × 10^7^ copies/container), and the oysters were exposed to the virus for 1 h under static conditions. After exposure, oysters in each treatment group were removed from the 100 mL container to tanks with running seawater as follows: 50 oysters were transferred to each of two 8 L aquaria containing 4 L of running seawater as the observation tanks. The remaining approximately 270 oysters were placed in a single 65 L aquarium containing 56 L of running seawater and used for sampling.

For the control, a group of 50 unexposed oysters was maintained under identical conditions in a separate observation tank. The observation tanks were monitored daily for mortality until 28 dpi. Oysters attached to the tank walls by byssus were counted as being alive, while oysters that had detached and settled on the bottom with loss of soft tissues were considered dead.

For sampling, eight live oysters were periodically collected from sampling tanks. The condition of their soft body was visually assessed through the semi-transparent shells under a stereomicroscope. After observation, the whole body of each oyster was homogenized in TRIzol LS (Invitrogen, Waltham, MA, USA), and total RNA was extracted. RNA concentration was determined by measuring absorbance at 260 nm using a Nivo Multimode Plate Reader (PerkinElmer, Shelton, CT, USA). PiBV copy number was quantified using reverse transcription-quantitative polymerase chain reaction (RT-qPCR) [16]. The total PiBV copy number per oyster was determined by multiplying the copy number per unit of template RNA by the total amount of RNA extracted from each individual.

### 2.4. Second Infection Experiment at HWT

Survivors from the LWT infection group in the first experiment (at 28 dpi) were pooled from the observation and sampling tanks and used for the second infection experiment. As a control, naïve juveniles maintained under LWT were used. The shell length of oysters based on 20 randomly selected individuals ranged from 6.1 to 12.0 mm (mean ± SD: 7.9 ± 1.6 mm). The water temperature for both the survivor and naïve groups gradually increased over three days to reach HWT, and oysters were acclimated for an additional 4 days in flowing seawater at HWT. At 35 dpi, three subgroups of 20 individuals from both the survivor and naïve groups were placed in six plastic dishes with 70 mm diameter containing 10 mL of autoclaved seawater at HWT. Two dishes in each group were infected with PiBV (8.7 × 10^6^ copies/dish), while one dish remained untreated as a control. After a 1 h static incubation at 25 °C, the oysters from each dish were transferred to separate 4 L aquaria with running HWT seawater and monitored daily for mortality for 22 days after the second infection.

Due to the limited number of oysters available, viral replication was indirectly assessed by quantifying PiBV in the tank effluent. A 100 mL aliquot of effluent was collected from each tank at regular intervals. FeCl_3_ solution (4.83 g of FeCl_3_∙6H_2_O in 100 mL of distilled water) was added to the seawater sample to reach a final concentration of 1.0 mg/L of ferric ion, the sample was stirred for 1 h at room temperature and then filtered under vacuum through a 0.8 μm polycarbonate membrane filter (Merck Millipore, Rahway, NJ, USA) to collect the virus aggregates. Total RNA was extracted from the filter using TRIzol LS, and PiBV was quantified by RT-qPCR [16]. Viral shedding (genome copies per minute per surviving individual) was calculated based on the flow rate (determined by the time required to collect 100 mL of effluent) and the number of surviving oysters in each tank. In preliminary tests (Appendix A), the recovery rate of PiBV using this method was approximately 30%.

### 2.5. Transcriptome Analysis

Since juvenile oysters exhibited resistance to reinfection, we hypothesized that basal gene expressions were involved. We performed comparative transcriptomic analysis using total RNA extracted from the whole bodies of survivors from the PiBV-infected group (*n* = 4) and the control group (*n* = 4) under LWT sampled at 28 dpi (Figure 1). The oysters (*n* = 8) had shell length ranging from 7.1 to 8.3 mm (mean ± SD: 7.7 ± 0.5 mm).

Total RNA was extracted using TRIzol LS and further purified with the NucleoSpin RNA XS kit (Takara Bio, Shiga, Japan). mRNA was isolated from total RNA using oligo(dT) beads and sequenced by BGI Genomics (Shenzhen, China). Prior to cDNA library construction, mRNA quality and concentration were assessed using a Bioanalyzer on the BGI platform. Paired-end sequencing (150 bp) was conducted using the DNBSEQ-T7. Raw sequencing reads were adapter-trimmed and quality-filtered by BGI Genomics, using SOAPnuke (version 1.5.2; https://github.com/BGI-flexlab/SOAPnuke (accessed on 22 February 2025)). In this process, reads matching ≥25.0% of the adapter sequence (maximum two base mismatches), reads with <150 bp of length, reads with ≥1.0% of N content, reads with >50 bp polyX, and reads with ≥40% of bases with <20 quality values were discarded. Clean reads were mapped to the reference genome *Pinctada fucata* ver. 3 [22] using CLC Genomics Workbench (GWB) ver. 25 (QIAGEN) (read mapping parameters: minimum fraction length of read overlap = 0.8, minimum sequence similarity = 0.8). Read counts for all genes were compared between the infected and control groups and normalized to counts per million (CPM) using the trimmed mean of M-values (TMM) normalization method [23] using the “Differential Expression in Two Groups” function with default parameters in CLC GWB. The similarities in gene expression patterns within each experimental group were confirmed by principal component analysis (PCA) of TMM-normalized CPM for all genes (no filtering) using the prcomp function with scaling (scale = TRUE) in R ver. 4.5.1 [24]. TMM-normalized CPM was calculated using the “Differential Expression in Two Groups” function with default parameters in CLC Genomics Workbench”.

Differentially expressed genes (DEGs) were defined as genes with an absolute log2 fold-change > 1 and a Benjamini–Hochberg false discovery rate (FDR)-corrected *p*-value < 0.05 between the infected and uninfected samples. The protein sequence of each gene was aligned against Swiss-Prot using DIAMOND blastp ver. 2.1.11 [25] and also searched using InterProScan ver. 5.73-104.0 [26]. The search results of DIAMOND blastp were imported to Blast2GO ver. 6.0.3, and gene ontology (GO) terms were assigned to each gene using the mapping function and merged with the InterProScan results using the annotation function in Blast2GO with default parameters [27]. The up-regulated (log2 fold-change > 1) and down-regulated (log2 fold-change < −1) DEGs were separately subjected to GO enrichment analysis using clusterProfiler ver. 4.16.0 in R [27,28]. Overrepresented GO categories associated with biological processes were filtered by the following conditions: (1) FDR-corrected *p*-value < 0.05; (2) the number of DEGs in the category > 5; (3) the number of genes in the category was between 10 and 500.

### 2.6. Ex Vivo Infection of Mantle Tissue Fragments

Mantle tissues from both naïve (*n* = 12) and previously infected (*n* = 12) juveniles were cultured ex vivo, and viral replication following PiBV infection was compared between the two groups. The naïve and previously infected oysters (*n* = 12 each) had shell length ranging from 9.0 to 19.0 mm (mean ± SD: 12.7 ± 3.1 mm) and from 9.0 to 20.1 mm (mean ± SD: 11.6 ± 2.5 mm), respectively. There was no significant difference in body size between the two groups (*p* = 0.1359, Welch’s *t*-test). Oysters in the naïve group were juvenile oysters in the control group of the second infection at HWT and previously infected juveniles were survivors of the first infection at LWT that were controls in the survivor group in the second infection experiment and were at 96 dpi at the start of this experiment (Figure 1). Both oyster groups were maintained at HWT after the second infection experiment.

Mantle tissue fragment culture was performed following methods of a previous study [18]. Briefly, one tissue fragment, including both the marginal and pallial zones, was excised from each of the left and right mantles and placed individually into wells of 48-well plates containing 300 μL of autoclaved seawater supplemented with 1000 units/mL of penicillin G and 1000 μg/mL of streptomycin sulfate (hereafter referred to as culture seawater). One of the two mantle fragments from each oyster was exposed to PiBV (1.7 × 10^5^ copies/well) at 25 °C for 24 h. After exposure, the tissue fragments were rinsed once with culture seawater and then put in 300 μL of fresh culture seawater at 25 °C. The other mantle fragment was cultured under the same conditions but without PiBV inoculation. On day 7 of ex vivo culture, 250 μL of seawater from each well was collected, RNA was extracted using TRIzol LS reagent, and PiBV was quantified using RT-qPCR.

### 2.7. Statistical Analysis

**Figure 2:**
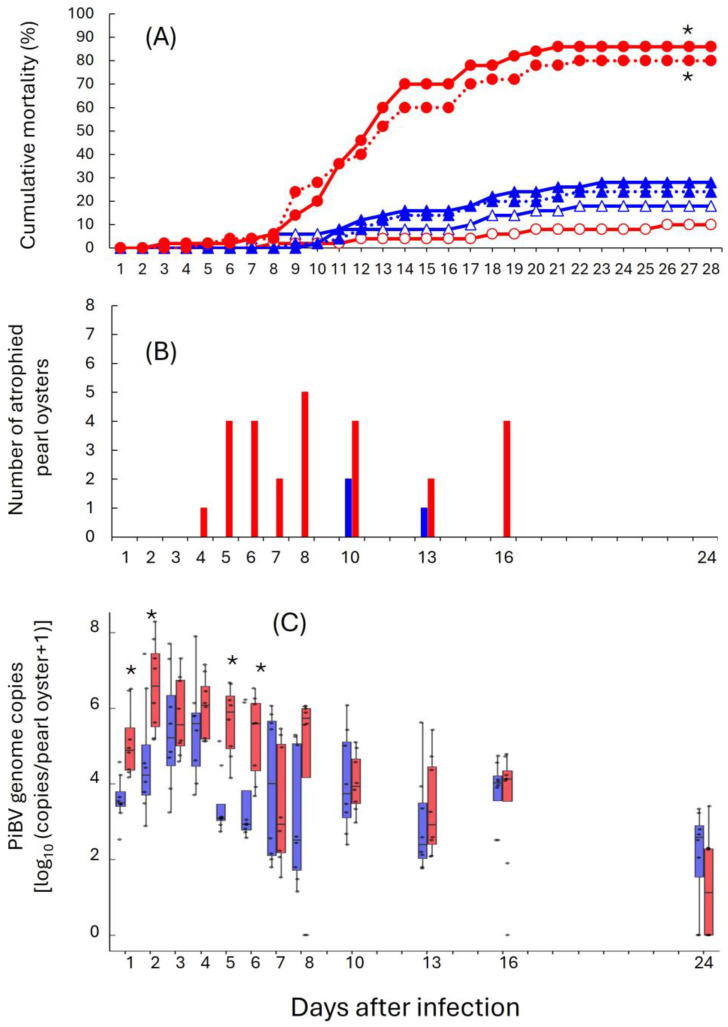
Results of the first infection experiment at different water temperatures. Red: High-water-temperature (HWT) groups. Blue: Low-water-temperature (LWT) groups. (**A**) Cumulative mortality. Closed and open symbols represent infected (duplicate tanks) and control (single tank) tanks, respectively. (**B**) Number of oysters with soft body atrophy in infected groups. Bars represent the number of oysters showing atrophy among 8 sampled oysters. Sampling and measurements were conducted only on the days indicated by the numerical labels on the *x*-axis. (**C**) PiBV genome copy numbers in individual oysters in infected groups. Error bars represent standard deviation. Asterisks indicate significant differences between LWT and HWT treatments (*p* < 0.05, Mann–Whitney U test on each sampling day). Sampling and measurements were conducted only on the days indicated by the numerical labels on the *x*-axis.

**Figure 3:**
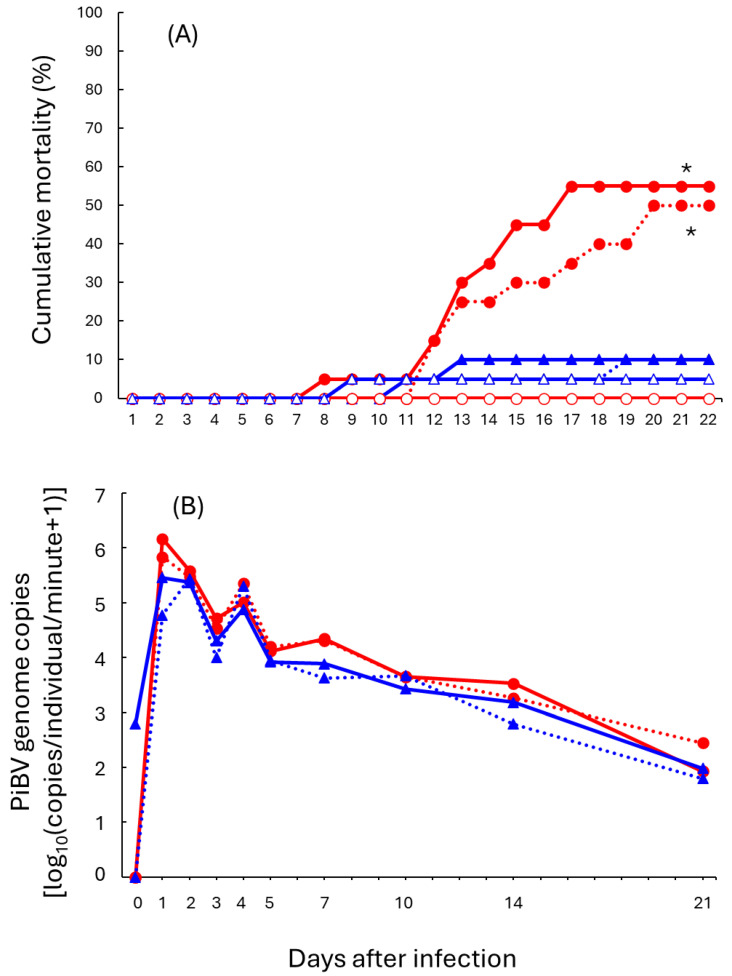
Results of second infection experiment at higher water temperature. Red: Reinfected groups; Blue: naïve groups. (**A**) Cumulative mortality. Closed and open symbols represent infected (duplicate) and control (single) tanks, respectively. Dashed lines represent duplicated tanks in infected groups. Asterisks indicate significant differences between first-time infection groups and all other treatment groups (*p* < 0.05, log-rank test with Benjamini–Hochberg correction). (**B**) PiBV genome copy number detected in tank effluent of infected groups. Virus shedding per oyster per minute was calculated based on the PiBV genome copies detected in 100 mL of effluent, the number of surviving oysters in each tank, and the water flow rate. Dashed lines represent a duplicated tank in each group.

Statistical analyses were performed using R ver. 4.5.1 [24]. Differences in survival curves between groups were assessed using the log-rank test, with multiple comparisons corrected by the Benjamini–Hochberg procedure (Figure 2A and Figure 3A). For the temporal changes in the number of atrophied individuals during the first infection experiment (Figure 2B), a generalized linear model (GLM) with a binomial distribution and a logit link function was applied. The response variable was the number of atrophied individuals out of eight oysters sampled at each time point, and the explanatory variables were rearing water temperature and sampling day. The statistical significance of each explanatory variable was evaluated using the Wald test. The results of PiBV genome copies quantification were expressed as mean ± SD after log_10_(X + 1) transformation. Normality and homogeneity of variance were assessed using the Shapiro–Wilk test and Levene’s test, respectively. To maintain consistency in the testing methodology and to ensure a conservative approach due to the small sample size, non-parametric tests were applied throughout, as some data groups did not meet the assumptions for parametric analysis even after transformation. The viral load between the two temperature groups at the same sampling time point (Figure 2C) was compared using the Mann–Whitney U test. In the second infection experiment, differences in viral load among tanks (Figure 3B) were tested using the Kruskal–Wallis test. For the ex vivo reinfection experiment using mantle tissue fragments (Figure 6A,B), statistical comparisons among groups were performed using Dunn’s test. A *p*-value less than 0.05 was considered statistically significant.

## 3. Results

### 3.1. First Infection Experiment at LWT and HWT

In the HWT group, cumulative mortality rates for the two infected observation tanks were 80% and 86%, which were significantly higher than those observed in other test groups (Figure 2A). In contrast, the two infected LWT observation groups had cumulative mortality rates of 24% and 28%, which were not significantly different from those in the uninfected control groups of LWT (18%) and HWT (10%).

From the sampled pearl oysters from the infected sampling groups, the number of oysters with atrophy among the eight individuals at each sampling is shown in Figure 2B. In the HWT group, between 1 and 5 atrophied individuals were observed from 4 to 16 dpi. In contrast, atrophy was observed in 1 or 2 oysters only at 10 and 13 dpi in the LWT group. The frequency of atrophy in the HWT-infected group was higher than in the LWT group, with water temperature as a significant explanatory variable in a Wald test.

Based on the number of PiBV genome copies detected in the eight sampled individuals, viral load peaked at 2 dpi for the infected HWT sampling group (Figure 2C). In the infected LWT sampling group, viral load peaked at 4 dpi. Comparison of viral loads between the two temperature groups at each time point showed significantly higher values in the infected HWT sampling group than in the infected LWT sampling group at 1, 2, 5, and 6 dpi. Viral load was thereafter low in both groups until 24 dpi, with PiBV undetectable in 2 out of 8 individuals in the LWT group and 4 out of 8 individuals in the HWT group.

### 3.2. Second Infection Experiment at HWT

The cumulative mortality rates of the naïve oyster groups exposed to PiBV were 50% and 55%, which were significantly higher than those of the other groups (Figure 3A). In contrast, the mortality rates of the groups of reinfected survivors were both 10%, showing no significant difference from the uninfected control groups (naïve oysters: 0%, survivors: 5%).

The number of PiBV genome copies detected in tank effluent peaked at 1 or 2 dpi. Although the naïve oyster groups tended to show higher viral shedding than the reinfected oyster groups, the difference was not statistically significant (Figure 3B).

### 3.3. Transcriptome Analysis

Mean total read count was 47.3 million (41.5 million to 48.2 million) per sample. All samples showed Q30 frequencies of bases higher than 97% and standard GC contents (40%), indicating good quality of the RNA sequencing reads. The percentages of paired mapped reads of total read counts ranged from 69% to 77%. Of the total 36,588 genes, DIAMOND blastp annotated 21,067 genes, InterProScan annotated 32,419 genes, and Blast2GO assigned GO terms to 18,071 genes.

**Figure 4:**
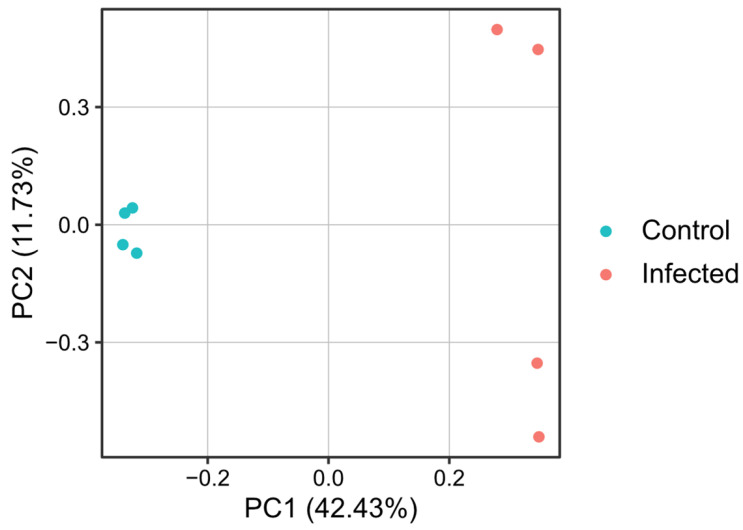
Principal component analysis (PCA) of total gene expression profiles in *Pinctada fucata*. TMM-normalized CPM of all genes in the control and the PiBV-infected groups was analyzed using the prcomp function in R.

Principal component analysis (PCA) based on TMM-normalized CPM of all genes demonstrated that the four individuals in the control group and those in the infected group formed distinct clusters, indicating that the two groups possessed different gene expression profiles (Figure 4).

**Figure 5:**
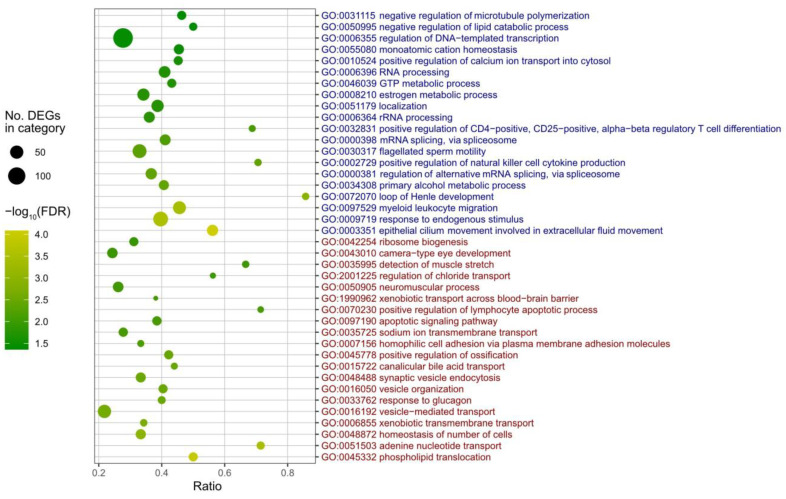
Results of GO enrichment analysis performed separately for up-regulated and down-regulated transcripts. For each group, the 20 most significantly over-represented GO terms were selected based on the lowest false discovery rate (FDR)-corrected *p*-values. Red and blue font colors indicate GO terms enriched among up-regulated and down-regulated genes, respectively. The *x*-axis represents the proportion of DEGs relative to the total number of genes associated with each GO term. Circle color denotes the log-transformed FDR-corrected *p*-value, and circle size corresponds to the number of DEGs assigned to each GO term. GO terms are ordered from bottom to top according to increasing FDR-corrected *p*-values (i.e., the smallest FDR-corrected *p*-value comes at the bottom).

Results of the GO enrichment analysis (biological process) of the DEGs that were down- or up-regulated in the infected group relative to the control group are presented in Figure 5 and Supplemental Table S1. The top 20 up-regulated GO categories with the lowest FDR-corrected *p*-values included processes related to substance transport (phospholipid translocation, adenine nucleotide transport, xenobiotic transmembrane transport, canalicular bile acid transport, sodium ion transmembrane transport, and xenobiotic transport across the blood–brain barrier), vesicular trafficking and organization (vesicle-mediated transport, vesicle organization, synaptic vesicle endocytosis), activation of apoptosis (apoptotic signaling pathway, positive regulation of lymphocyte apoptotic process), activation of muscle and neuronal functions (neuromuscular process, detection of muscle stretch), hormonal responses (response to glucagon), and bone metabolism (positive regulation of ossification). The top 20 down-regulated GO categories with the lowest FDR values included processes associated with RNA processing (regulation of alternative mRNA splicing via the spliceosome, mRNA splicing via the spliceosome, rRNA processing, and RNA processing) and transcription (regulation of DNA-templated transcription), immune responses (myeloid leukocyte migration, positive regulation of NK cell cytokine production, and positive regulation of CD4^+^ CD25^+^ αβ Treg differentiation), metabolism (primary alcohol metabolic process, estrogen metabolic process, and GTP metabolic process), and cell motility (epithelial cilium movement, localization, and negative regulation of microtubule polymerization).

### 3.4. Ex Vivo Infection Experiment on Mantle Tissue Fragments

**Figure 6:**
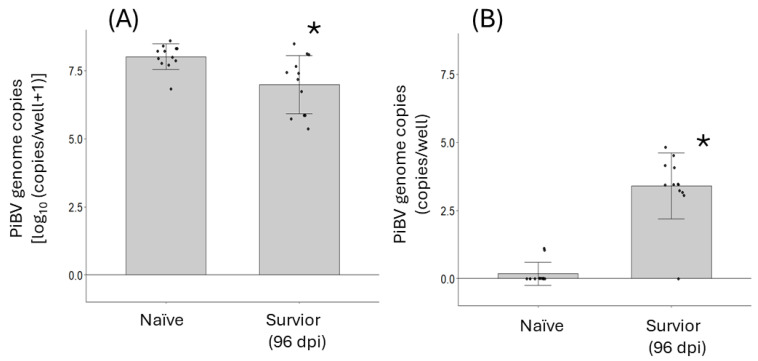
PiBV genome copy numbers in culture supernatants of mantle tissue fragments cultured ex vivo with or without PiBV inoculation. (**A**) Mantle fragments inoculated with PiBV ex vivo. (**B**) Mantle fragments cultured without PiBV inoculation. Naïve: Juvenile pearl oysters with no prior exposure to PiBV. Survivor: Juveniles that were exposed at LWT in the first infection experiment and survived at 96 days post-infection (dpi). Each dot represents data from an individual oyster. Asterisks indicate significant differences (*p* < 0.05, Mann–Whitney U test).

After ex vivo infection with PiBV, the amount of viral genome detected in culture supernatant was significantly higher in mantle fragments from naïve oysters than in survivors at 96 dpi from the first infection at LWT (Figure 6A). For mantle tissues cultured ex vivo without PiBV inoculation, the culture supernatant from survivors contained significantly higher amounts of PiBV genome than that from naïve oysters (Figure 6B). The detection rate of PiBV in the culture supernatants without PiBV inoculation was 16.7% for the naïve group and 91.7% for the survivor group. The PiBV genome copy number detected in the naïve group (Ct = 35.9–36.8) was close to the y-intercept of the standard curve (Ct = 37.1), suggesting that these low-level signals may represent minor contamination or non-specific amplification.

## 4. Discussion

Consistent with previously reported immune-like phenomena observed under natural conditions [19,20], juvenile pearl oysters that had experienced a prior PiBV infection exhibited higher survival rates upon reinfection (Figure 3A). This difference in survival rates is unlikely to have resulted from the selection of inherently resistant individuals during the first infection experiment, because the cumulative mortality of the infected group at LWT did not differ significantly from that of the uninfected control group (Figure 2A). Therefore, no selective survival advantage was expected in the reinfection experiment. The observed disease resistance is also unlikely to be attributable to tissue damage caused by the first infection. PiBV infects the outer epithelial cells of the mantle, but tissue damage caused by the disease is rapidly repaired. Histopathological analysis has shown that while exfoliation of mantle epithelial cells was observed at 2 dpi, when viral load was at its peak, the tissue had already recovered by 4 dpi, when the viral load had decreased, and no histological abnormalities were detectable [16]. Moreover, individuals with soft-body atrophy collected from aquaculture farms showed no histopathological abnormalities, including those in the mantle epithelium [15,16]. The timing of reinfection, at 28 dpi, makes it unlikely that tissue-level alterations, such as the loss of target cells, would contribute to disease resistance. Although body size can influence mortality in summer atrophy [15], there were no apparent or systematic differences in size between our experimental groups. Given this consistency, it is unlikely that the observed variations in survival were attributable to differences in body size. Therefore, the resistance to reinfection observed in this study is considered to be a result of immune priming—that is, the ability of the innate immune system to mount an enhanced response to a subsequent infection following prior exposure [29].

However, several experimental constraints must be acknowledged when interpreting these results. While our data is consistent with an immune memory-like response, the limited sample sizes and inter-individual variability may constrain the strength of our mechanistic inferences. Specifically, since viral quantification in the second infection experiment was based on shedding into the water rather than direct tissue titration (Figure 3B), these values provide only an indirect measure of viral replication. Furthermore, it is possible that persistent, low-level infection (Figure 6B) plays a role in maintaining a heightened defensive state through continuous immune stimulation, rather than through a classic “memory” mechanism (see the later discussion regarding persistent infection).

Immune memory-like responses against PiBV in the pearl oyster appear to operate in the mantle, the local site of infection, with at least sufficient efficacy to significantly suppress PiBV replication. Mantle tissues from naïve oysters cultured ex vivo and infected with PiBV showed significantly higher amounts of viral genome in the culture supernatant than those from previously infected oysters (Figure 6A), suggesting that the immune memory-like response in *P. fucata* may not require specialized immune organs such as hematopoietic or lymphoid tissues as in vertebrates. However, because ex vivo-cultured tissue fragments contain hemocytes and hemolymph components, the involvement of these peripheral defense factors in the immune response cannot be ruled out. Moreover, for infection trials using living oysters, the possibility that tissues other than the mantle also contribute to protection against reinfection cannot be excluded. Therefore, whether the immune-like phenomena in *P. fucata* is entirely localized or whether there are organs with functionality similar to vertebrate lymphoid tissue remains to be determined. Further studies, such as verifying immune responses using mantle outer epithelial cell cultures or conducting cell/tissue transplantation experiments, are needed to clarify this point.

Even slight differences in viral replication may be a factor in determining the survival or death of the oysters. In the reinfection experiment conducted under HWT, groups infected for the first time tended to show slightly higher viral shedding compared to the re-infected group, although these differences were not statistically significant (Figure 3B). The maximum viral shedding per individual during the observation period was approximately two to five times higher in the first infection groups than in the reinfection group. Similarly, in the ex vivo infection experiment, the mean viral genome copy number in the culture supernatant of naïve mantle tissues was about twice that of the survivor (96 dpi) group (Figure 6A). In infection experiments conducted at different water temperatures, a comparison between HWT (with >80% mortality) and LWT (with <30% mortality) revealed only a four-fold difference in the maximum PiBV load during the observation period (Day 2 for HWT and Day 4 for LWT; Figure 2C), despite significantly different mortality rates. These several-fold differences represent a relatively narrow margin in terms of viral dynamics. These findings suggest that even modest suppression of viral replication might potentially reduce the impact of this disease, offering a promising prospect for future management.

Most infected oysters remained carriers of PiBV for approximately 3 months, and persistent infection with PiBV may stimulate the immune system, potentially inducing an immune-like phenomenon. On the other hand, in the quantification of PiBV in infected individuals, viral genomes were undetectable in 2 of 8 oysters at 24 dpi for treatment at LWT and in 4 of 8 oysters at HWT (Figure 2C). We interpret these results as reflecting viral loads falling below the detection limit of RT-qPCR in vivo, rather than indicating complete clearance. However, we interpret them as viral loads falling below the detection limit of RT-qPCR in vivo. This interpretation is strongly supported by our ex vivo experiments. When mantle tissues from survivors at 96 dpi were cultured without the addition of PiBV, viral genomes were recovered in the culture supernatant from 11 out of 12 individuals (Figure 6B). Although the viral loads in the supernatant, to which no virus was added, remained relatively low (Ct values ranging from 29.19 to 36.83), the consistent detection of viral genomes after ex vivo incubation provides evidence that the virus persists in a viable state capable of limited replication or release. While we acknowledge the sensitivity limits of RT-qPCR, these results suggest that low-level persistent infection could potentially stimulate the oyster’s immune system to induce immune memory-like responses. Although summer atrophy ceases when water temperatures drop in autumn and winter, the disease has recurred in the same farmed areas every summer since its first outbreak in 2019. PiBV did not replicate in other tested mollusk species, including the Pacific oyster (*M. gigas*), the Manila clam (*Ruditapes philippinarum*), the blue mussel (*Mytilus galloprovincialis*), and an abalone (*Haliotis discus discus*), indicating host specificity (unpublished data). The annual recurrence of PiBV disease in affected areas is therefore likely caused by reactivation of the virus persisting in previously infected *P. fucata*.

Transcriptome analysis of juvenile oysters at 28 dpi revealed distinct transcriptional responses between the infected and naïve groups (Figure 4 and Figure 5). Genes associated with GO categories related to substance transport and vesicle trafficking, activation of apoptosis, activation of muscle and neuronal functions, and bone metabolism were upregulated, whereas genes associated with immune responses, RNA processing and transcription, metabolism, and cell motility were downregulated. Thus, at 28 dpi, oysters appear to exhibit enhanced cell death, turnover of cellular components, muscle and neuronal activity, and shell formation, while immune responses and basic metabolic activity are suppressed. These conditions may contribute to producing a defensive state against PiBV reinfection. However, as described in the following paragraph, GO terms associated with antiviral responses—such as those related to interferon-like pathways—showed no appreciable changes, and therefore the present analysis did not clarify the immune response that confers resistance to PiBV.

In the Pacific oyster (*M. gigas*), field studies have shown that individuals previously exposed to OsHV-1 exhibit higher survival rates during subsequent outbreaks [30]. Experimental studies have also demonstrated that prior infection with OsHV-1 [11,31,32], exposure to inactivated OsHV-1 [12,33], and administration of synthetic double-stranded RNA (dsRNA) analogs such as poly(I:C) [9,10,34,35,36] can enhance resistance to OsHV-1 infection. The antiviral protection induced by dsRNA treatment in Pacific oysters appears to be much stronger than the immune-like response observed in pearl oysters against PiBV. As mentioned above, in the PiBV reinfection experiments, the amount of virus released from pearl oysters and from ex vivo-cultured mantle tissues decreased only to a fraction of the levels observed during the primary infection. In contrast, in Pacific oysters challenged with OsHV-1 after poly(I:C) administration, viral genome copy numbers were reduced by several hundred-fold [9,10]. The protection induced by prior OsHV-1 exposure or poly(I:C) stimulation has been attributed to the sustained upregulation of genes involved in the interferon-like and NF-κB signaling pathways (including receptors such as RLR, the adaptor protein MyD88, transcription factors IRF, and antiviral effectors such as ADAR and viperin), apoptosis-related genes (IAP), and autophagy-related genes (ATG8 and Beclin) [12,33,34,36,37,38,39]. Because PiBV possesses a double-stranded RNA genome, it is reasonable to assume that it could also trigger antiviral immune pathways like those stimulated by poly(I:C) stimulation. However, in the present study, upregulation of these genes, except for ATG8, was not observed (Appendix B). Moreover, upregulation of genes related to the NF-κB signaling pathway, Notch signaling pathway, Jak/STAT signaling pathway, Th17 cell differentiation, cell adhesion molecules, and peroxisome-related genes reported in *P. fucata* after poly(I:C) injection [40], as well as increased expression of Toll-like receptor genes [41], were not detected in our analysis. These discrepancies may be a result of using whole-body samples for the expression analysis, which could have masked important immune responses occurring in rare, localized cell populations within the overall RNA signal. Specifically, the inclusion of tissues unrelated to the immune-like response may have diluted the transcriptomic signals originating from the mantle, the primary site of infection. Alternatively, the absence of upregulation in these genes may be attributed to the relatively long period between infection and sampling (28 dpi) in the present study, compared with the *M. gigas* study, in which samples were collected 24 h after OsHV-1 inoculation [12]. It is also possible that, unlike in Pacific oysters, an interferon-like response may not occur in pearl oysters upon PiBV infection, which could explain why a strong protective effect was not obtained in this case compared to that observed in OsHV-1 infection tests following poly(I:C) administration. Despite these potential factors, sampling at 28 dpi was strategically intended to identify changes in basal gene expression that might be responsible for the acquired resistance observed in our reinfection experiments. By focusing on this later time point, we sought to capture a stable physiological state rather than a transient response to acute infection. To better understand the immune-like responses of *P. fucata*, it will be essential to perform expression analyses on mantle tissue, where the immune response against reinfection is exerted, or on the outer mantle epithelial cells, which are the primary target of PiBV. Furthermore, evaluating the effects of poly(I:C) administration in pearl oysters should be a focus of future studies.

Infections at LWT resulted in lower PiBV replication, less frequent occurrence of soft-tissue atrophy, and lower mortality rate compared with infections at HWT (Figure 2). Hashimoto et al. [19] similarly reported that both mortality and the incidence of soft-tissue atrophy decreased as water temperature decreased. In pearl culture fields, PiBV-infected pearl oysters are typically detected when seawater temperatures exceed 20 °C, yet mortality rates at this temperature are not high [19]. In the HWT infection experiment, the rapid proliferation of PiBV may have caused severe damage to the mantle tissue, leading to a higher incidence of soft-tissue atrophy and increased mortality. Most diseases in poikilothermic animals become severe only within specific temperature ranges. Therefore, a strategy of enhancing immune function by exposing invertebrates to pathogens outside the temperature range in which disease develops may be effective for preventing infectious diseases. Actually, *M. gigas* exposed to OsHV-1 at 18 °C, a temperature that does not cause high mortality, showed reduced mortality when reinfected at 22 °C, where mortality is normally high [32]. To apply such an approach in aquaculture, however, it will be essential to clarify the duration of protection and safe exposure conditions, as well as to evaluate potential risks, such as the spread of pathogens to unaffected areas through the movement of carrier organisms and the possible emergence of viral variants due to increased opportunities for viral replication.

## 5. Conclusions

Prior infection with PiBV in the pearl oyster *P. fucata* enhances survival and suppresses viral replication upon reinfection, supporting the existence of immune priming, as seen in other invertebrates. Ex vivo experiments showed reduced PiBV replication in mantle tissues derived from previously infected oysters, suggesting the involvement of a localized defensive mechanism. Transcriptome analysis did not reveal clear activation of antiviral pathways such as interferon-like responses, implying that resistance to PiBV in *P. fucata* may rely on mechanisms distinct from those reported for OsHV-1 resistance in the Pacific oyster. Although the differences in viral load observed in this study were relatively small, they were sufficient to influence survival, indicating that even modest suppression of viral replication may reduce disease impact.

Water temperatures strongly affect mortality, and non-lethal infection at lower temperatures may provide a basis for inducing protective responses. Together, these findings provide important insights into PiBV pathogenesis and offer a foundation for developing novel disease management strategies in pearl oyster aquaculture.

## Figures and Tables

**Figure A1:**
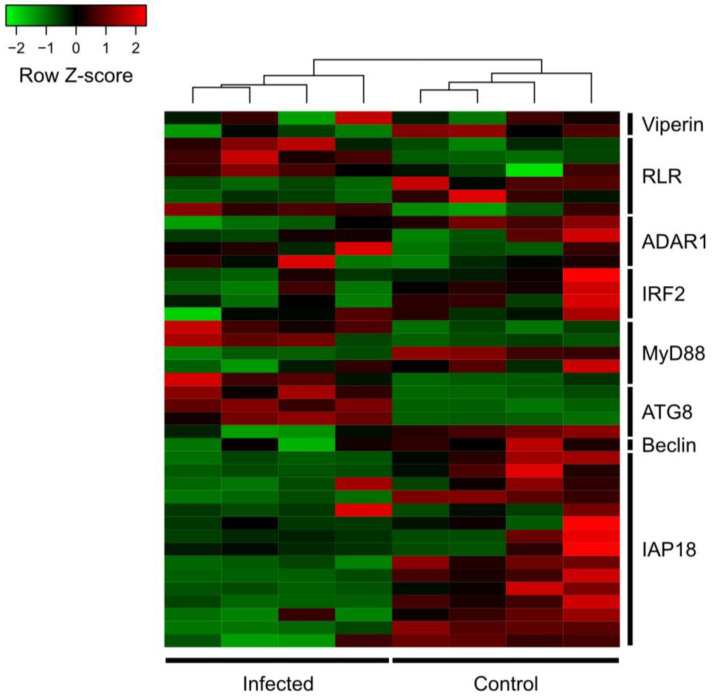
Heat map showing expression levels of *P. fucata* genes homologous to the eight candidate genes reportedly associated with antiviral responses to OsHV-1 in *M. gigas* [12]. The z-scaled gene expressions are shown on the heat map.

## Data Availability

The sequence data generated in this study have been deposited in the DDBJ database. All accession numbers are listed in Supplementary Table S2.

## Supplementary Materials

The following supporting information can be downloaded at: https://www.mdpi.com/article/10.3390/pathogens15040380/s1.

## Abbreviations

The following abbreviations are used in this manuscript:

PiBV	Pinctada birnavirus
OsHV-1	Ostreid herpesvirus 1
HaHV-1	Haliotid herpesvirus 1
HWT	Higher water temperature
LWT	Lower water temperature
RT-qPCR	Reverse transcription-quantitative polymerase chain reaction
CPM	Read counts per million
TMM	Trimmed mean of M-values
PCA	Principal component analysis
DEGs	Differentially expressed genes
FDR	False discovery rate
GO	Gene ontology
dpi	Days post-infection

## Appendix A. Verification of PiBV Concentration Efficiency in Seawater Using Iron Flocculation Method

Objective

To evaluate whether PiBV in seawater can be concentrated and quantified using an iron flocculation method, in which negatively charged viral particles in water are aggregated with ferric ions and collected on a filter [42].

Materials and Methods

A PiBV suspension of known concentration was added to 1 L of autoclaved seawater to prepare a viral suspension containing 4.05 × 10^6^ copies per 300 mL. Ferric chloride solution (FeCl_3_·6H_2_O, 4.83 g dissolved in 100 mL of distilled water) was added to the suspension to achieve a final ferric ion concentration of 1.0 mg/L. The mixture was stirred at room temperature for 1 h and then 300 mL was filtered through three 0.8 μm pore-size polycarbonate filter (Millipore) under reduced pressure to capture the viral aggregates. RNA was extracted from each filter using TRIzol LS reagent, and the amount of PiBV was quantified by RT-qPCR [16]. The viral copy number per 300 mL of seawater was calculated.

Results

In three independent filtration trials, the PiBV genome copy numbers were determined to be 1.20 × 10^6^, 1.16 × 10^6^, and 1.29 × 10^6^ copies per 300 mL, corresponding to approximately 30% recovery of the initially added viral amount.

## Appendix B. Transcriptomic Assessment of OsHV-1–Related Antiviral Genes in PiBV Infection

Objective

Previous research [12] reported that eight genes are up-regulated during the antiviral response to OsHV-1 in Pacific oysters. In this study, we examined transcriptomic data to determine whether these genes also show expression changes during the immune memory-like response of the pearl oyster to PiBV.

Materials and Methods

Genes homologous to the eight previously analyzed *Magallana gigas* genes [12] (Table A1) were searched in the *Pinctada fucata* genome ver. 3 [22] using BLASTp (ver. 2.16.0). Expression data of these genes were extracted from the transcriptome data obtained in this study. Genes with low expression (CPM ≤ 5 in all samples) were excluded from subsequent analysis. CPM of each gene was Z-score normalized among samples. Z-scaled gene expression was visualized on a heatmap using the heatmap.2 function in the gplots ver. 3.2.0 in R with clustering among samples (Warnes MGR, Bolker B, Bonebakker L, Gentleman R. Package ‘gplots.’ Var R Program Tools Plotting Data 2016).

**Table A1:** *Magallana gigas* genes used in the analysis.

Name	Accession No.	Function
viperin	EKC28205	IFN pathway
RLR	EKC34573	IFN pathway
ADAR1	EKC20855	IFN pathway
IRF2	EKC43155	IFN pathway
MyD88	DQ530619.1	NF-kB pathway
ATG8	EKC40439.1	autophagy
Beclin	EKC28450.1	autophagy
IAP18	JH818926	apoptosis

Results and Discussion

Homology analysis identified 2, 6, 4, 5, 6, 4, 1, and 13 homologs corresponding to *M. gigas* viperin, RLR, ADAR1, IRF2, MyD88, ATG8, Beclin, and IAP18, respectively. Among the four homologous genes corresponding to ATG8 in *M. gigas*, three genes exhibited a consistent tendency to be up-regulated in all four infected individuals (Figure A1). The one gene that did not show up-regulation had markedly lower expression levels than the others and showed the lowest sequence similarity to the *M. gigas* reference. For the other *M. gigas* genes, while some *P. fucata* homologs, such as those related to RLR and MyD88, displayed a tendency toward up-regulation in the infected group, no homolog group showed a consistent up-regulation pattern among its members.
